# Magnetic Drug Delivery: Where the Field Is Going

**DOI:** 10.3389/fchem.2018.00619

**Published:** 2018-12-11

**Authors:** Paige M. Price, Waleed E. Mahmoud, Ahmed A. Al-Ghamdi, Lyudmila M. Bronstein

**Affiliations:** ^1^Department of Chemistry, Indiana University, Bloomington, IN, United States; ^2^Department of Physics, Faculty of Science, King Abdulaziz University, Jeddah, Saudi Arabia; ^3^A.N. Nesmeyanov Institute of Organoelement Compounds, Russian Academy of Sciences, Moscow, Russia

**Keywords:** magnetic drug delivery, magnetic targeting, theranostics, iron oxide, magnetic implant

## Abstract

Targeted delivery of anticancer drugs is considered to be one of the pillars of cancer treatment as it could allow for a better treatment efficiency and less adverse effects. A promising drug delivery approach is magnetic drug targeting which can be realized if a drug delivery vehicle possesses a strong magnetic moment. Here, we discuss different types of magnetic nanomaterials which can be used as magnetic drug delivery vehicles, approaches to magnetic targeted delivery as well as promising strategies for the enhancement of the imaging-guided delivery and the therapeutic action.

## Introduction

The majority of anticancer drugs are delivered intravenously and accumulated in tumors containing the abundance of leaking blood vessels. However, this affects healthy tissue and causes numerous side effects. The more efficient approach is realized when drug nanocarriers are functionalized with target molecules [for example, folate (FA) groups], which interact with specific receptors located in certain tumors, allowing for the attachment of the drug delivery vehicles solely to the tumor (Fernandez et al., 2018; Rosiere et al., 2018; Sun, Q. et al., 2018; Sun, W. et al., 2018). This approach allows for a significant decrease of side effects caused by chemotherapy agents (Li et al., 2017; Peng et al., 2017; Sun, W. et al., 2018). Another drug delivery approach which can be used for many types of tumors is magnetic drug targeting which can be achieved if a drug delivery vehicle possesses a strong magnetic moment and can be manipulated by a magnetic field (Lee et al., 2017; Luong et al., 2017; Wei et al., 2017).

Magnetic drug delivery was first introduced in the 80's (Widder et al., 1980; Kost and Langer, 1986) but in the last decade the interest to magnetic targeting soared due to the development of stronger magnets and higher sophistication magnetic probes with multiple functions, i.e., theranostic probes (Nan et al., 2017; Sun, Q. et al., 2018; Tang et al., 2018). Such probes allow for a combination of diagnostics (magnetic resonance imaging (MRI) or magnetic particle imaging), and therapeutics, which could include hyperthermia and drug release as well as targeted drug delivery (for example, with an applied magnetic field).

A substantial number of reviews has been published on magnetic drug delivery (Kost and Langer, 1986; Lubbe et al., 2001; Duran et al., 2008; Herrmann et al., 2009; Williams et al., 2009; Foy and Labhasetwar, 2011; Tietze et al., 2012, 2015; Mody et al., 2014; Lyer et al., 2015; Mitra et al., 2015; Shapiro et al., 2015), the latest of which appeared as recently as 2016–2017 (Ulbrich et al., 2016; Grillone and Ciofani, 2017; Kralj et al., 2017; Mosayebi et al., 2017). However, the explosive development of this field in the last two years reveals the need in reviewing recent findings and better understanding of the major trends and shortcomings.

## Development of Magnetic Drug Delivery Probes

Currently, there are many different types of magnetic bioprobes which are being explored for magnetic targeting. In this review, we will focus on the most promising bioprobes from the viewpoint of magnetic manipulation and loading/release of specific drugs.

### Magnetic Nano/Microparticles

Magnetic microspheres were developed to overcome two major issues that are present with non-magnetic microcarriers: reticuloendothelial system clearance and poor site specificity (Kakar et al., 2013). One of the approaches is to develop porous or hollow/porous microspheres from magnetic spinel ferrites M_x_Fe_3−x_O_4_ (M = Fe, Zn). Their high magnetism means the microspheres can be easily manipulated by a magnet within the vascular system and, more specifically, remain in the target organ capillaries. Chen et al. utilized a hollow nanoparticle (NP) with a mesoporous shell which creates a higher surface area and a large cavity where drug can be encapsulated in both the mesopores and the cavities (Chen et al., 2017). Additionally, M_x_Fe_3−x_O_4_ (M = Fe, Zn) produce more heat under microwave irradiation which allows easier release of the loaded drug. However, doping of iron oxide causes the decrease of the saturation magnetization which diminishes the microsphere potential for magnetic targeting (Chen et al., 2017).

Another approach to synthesizing microspheres is the combination of a polymer with inorganic NPs. Wang et al. utilized poly(ε-caprolactone) (PCL) to encapsulate both Fe_3_O_4_ NPs and the anti-cancer drug, doxorubicin hydrochloride (DOX) (Wang, G. et al., 2018). The superparamagnetic composite microspheres showed a high drug loading and a quick magnetic response. The drug release was shown to be pH-sensitive with a high initial release and sustained release over many days.

Microparticles of dry powder chemotherapeutic containing iron oxide NPs (called nano-in-microparticles, NIMs) were used for magnetic delivery into lungs with an applied magnetic field (Price et al., 2017). Mice were endotracheally administered fluorescently labeled NIMs as a dry powder in the presence of an external magnet placed over one lung. It was demonstrated that in the magnetically activated lung, DOX loaded NIMs were therapeutically efficient, thus allowing for a targeted delivery.

Specific gene delivery has been realized with biomimetic magnetic microparticles (magnetosomes) synthesized utilizing magnetic nanocluster (MNC) core and Arg–Gly–Asp (RGD) decorated macrophage shell (Zhang et al., 2018). The magnetosome synthesis was accomplished via several steps including MNC preparation, azide-membrane engineering, electrostatic assembly, and click chemistry. This complex approach to magnetosomes is well-justified, allowing for high-performance siRNA delivery through a superior stealth effect, MRI, magnetic accumulation via an external magnetic field, RGD targeting, and favorable cytoplasm trafficking.

Drug-loaded microparticles prepared by layer-by-layer (LbL) deposition of polyelectrolytes with embedded magnetic NPs were attached to *Escherichia coli* bacteria, creating stochastic “microswimmers” which moved at average speeds of up to 22.5 μm/s (Park et al., 2017). These “microswimmers” displayed biased and directional motion under a chemoattractant gradient and a magnetic field, respectively. This work demonstrates that multifunctional bacteria-driven magnetic bioprobes can be used for targeted drug delivery with significantly enhanced drug transfer in comparison to passive microparticles. Another interesting example of “microswimmers” was reported in (Stanton et al., 2017). The non-pathogenic magnetotactic bacteria Magnetosopirrillum gryphiswalense (MSR-1) was combined with antibiotic loaded mesoporous silica microtubes for targeting an infectious biofilm. Combining magnetic guidance property and swimming power of the MSR-1 cells, the biocomposite particles have been delivered to the matured *E. coli* (*E. coli*) biofilm followed by the antibiotic release and the biofilm disruption, revealing a potential for antibiofilm applications.

**Figure 1:**
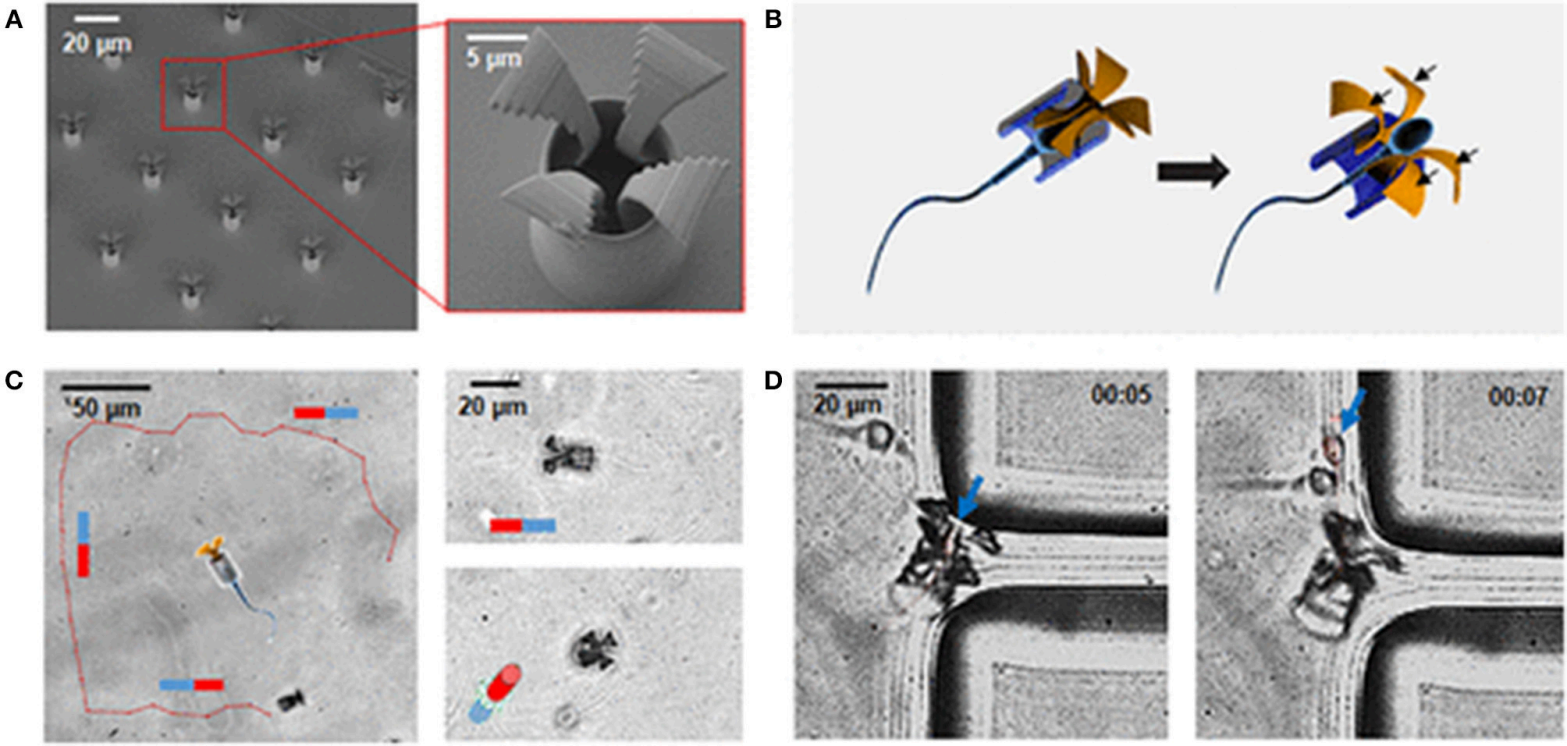
**(A)** SEM images of an array of printed tetrapod microstructures. **(B)** Schematic illustrating the mechanical release mechanism. **(C)** Track (red line) of a sperm-hybrid motor under magnetic guidance in the horizontal and vertical planes. **(D)** Image sequence of a sperm release process when the arms hit the corner of a polydimethylsiloxane wall. Blue arrows point at the sperm head. Time lapse in min:s (Xu et al., 2018). Reproduced with the permission of the copyright holder [American Chemical Society].

Xu et al. reported the development of an unprecedented sperm-hybrid micromotor for targeted drug delivery (Xu et al., 2018). This micromotor consists of a motile sperm cell which is both a propulsion source and a drug carrier (Figure 1). The other component is a 3D-printed magnetic tubular microstructure (called “tetrapod”) made of a polymer and coated with 10 nm Fe and 2 nm Ti (to protect Fe from oxidation). The tetrapod contains four arms which release the sperm cell *in situ* when they are pushed into a tumor. A magnetic field allows for controllable magnetic guidance as well as release, allowing drug delivery to tumor cells without damaging the healthy tissue. This system combines high drug loading capacity, self-propulsion, *in situ* mechanical trigger release of the drug-loaded sperm, sperm penetration ability, and improved drug availability.

In order to develop multifunctional NPs combining a near-infrared (NIR) plasmonic response with magnetic targeting, Tsai et al. deposited a double layer of Au/Ag alloy on the surface of truncated octahedral iron oxide NPs (Tsai et al., 2018). The rattle-shaped nanostructures exhibited a response for photothermal therapy (PTT) and magnetic guidance for hyperthermia and MRI as well as accumulation of the probes using an external magnetic field. The distance between the layers was controlled for maximum NIR absorption. These probes do not require a drug for chemotherapy as a dual action is already realized with PTT and hyperthermia.

### Nanoparticle Clustering/Assembly

One of the primary issues with using superparamagnetic iron oxide NPs is that the individual NPs do not display high magnetization which is unfavorable for guiding them through the body (Kralj et al., 2017). A way to overcome this problem is clustering of NPs to increase their overall magnetic response. Zheng et al. synthesized copolymers of hyaluronic acid (HA) and C_16_ micelles using peptide formation followed by encapsulation of docetaxel (DTX, an anti-cancer agent) and NPs to develop multifunctional micelles (Zheng et al., 2018). HA is especially attractive because it binds to the CD-44 receptor which is overexpressed in various types of cancer in addition to its biocompatibility and biodegradability (Lee et al., 2012). Cellular uptake occurred via CD-44 receptor-mediated endocytosis and was enhanced by the presence of a magnetic field. This uptake method increases the amount of micelles in the tumor tissues compared to the normal tissues that creates a favorable contrast in MR images. Furthermore, drug release was triggered by NIR irradiation.

Assembly of iron oxide NPs on polydopamine (PDA) NPs allowed for an enhanced magnetic response and stimuli-controlled drug release for *in vivo* cancer theranostics (Ao et al., 2018). The high reactivity of the PDA surface furnishes a possibility for reduction-responsive prodrugs, while poly(ethylene glycol) (PEG) chains allow for *in vivo* cancer therapy. Due to a combination of MRI/photoacoustic dual-modal tumor imaging and controlled magnetic drug targeting, the effective tumor obliteration was accomplished via synergy of NIR photothermal ablation (due to PDA) and anticancer drug magnetic delivery.

Iron oxide NPs with the grafted poly(styrene)-*b*-poly(acrylic acid) (PS-*b*-PAA) block copolymer were self-assembled into multilayer magneto-vesicles (MVs) and utilized for incorporation of drugs in the hollow cavity (Yang et al., 2018). High packing density of iron oxide NPs in MVs allowed for the high magnetization and transverse relaxivity rate (*r*2) in MRI. When injected, DOX-loaded MVs conjugated with RGD peptides were efficiently accumulated in tumor cells due to magnetic targeting.

Innovative magnetic drug delivery vehicles were developed based on magnetite NP clusters (Wang, Y. et al., 2018). Two shells were built on the NP cluster surface: an inner shell of PDA with attached triphenylphosphonium (TPP) and an outer shell of methoxy PEG (mPEG) linked to PDA by disulfide bonds. At the first stage of targeting, the magnetic NP clusters allow for the bioprobe accumulation at the tumor site using an external magnetic field. At the second stage, mitochondrial targeting takes place as the mPEG shell is removed from the NPs by a redox reaction to expose TPP. Upon NIR irradiation at the tumor site, a photothermal effect is produced from the PDA photosensitizer, resulting in a strong decrease in mitochondrial membrane potential. At the same time, DOX is released, leading to the damage of mitochondrial DNA and cell death. Thus, photothermal therapy and chemotherapy are combined with magnetic drug delivery resulting in an enhancement of the DOX performance.

### Magnetic Microbubbles

Image-guided and targeted modulation of drug delivery by external physical triggers at the site of pathology has been promising for drug targeting (Vlaskou et al., 2010; Cai et al., 2012). Magnetic microbubbles (MagMB) that are responsive to magnetic and acoustic field changes and visible to ultrasonography were suggested for magnetic drug targeting. Recently, MagMB were prepared by mixing the suspension of protamine-functionalized microbubbles (MB-Prot) with the suspension of the heparinized NPs (Chertok and Langer, 2018). MagMB were gathered in tumor by magnetic targeting and observed by ultrasonography. Using focused ultrasound, MagMB were collapsed when necessary to release a drug. The authors demonstrated drug delivery to tumors could be enhanced by optimizing magnetic and acoustic fields, using ultrasonographic monitoring of MagMB in real-time.

Tang et al. synthesized multifunctional MagMB utilizing poly(lactic-*co*-glycolic acid) (PLGA), FA, perofluorohexane (PFH), Fe_3_O_4_, and DOX (Tang et al., 2018). These nanocomposites are able to move intravenously due to their initial nanometer-range size. However, when high-intensity focused ultrasound (HIFU) is used, PFH is transformed from the liquid to the gas phase due to an increase in temperature. The PFH gas forms microbubbles which enhance the ultrasound image. Fe_3_O_4_ allows for the nanocomposite to be efficiently targeted through MRI guidance. Additionally, the FA ligands on the surface of the nanocomposites specifically target the tumor cells via conjugation. The DOX release is triggered by HIFU exposure, and the release is accelerated due to the tumor-acidic microenvironment. Finally, Fe_3_O_4_ NPs enhance the sensitivity of the tumor via the hyperthermia effect (Moroz et al., 2001). This nanocomposite is an efficient and comprehensive theranostics probe.

Another multifunctional MagMB are based on magnetic liposomes (Liu et al., 2017a). Liposomes range in size from 50 to 1,000 nm and are biocompatible. Both water-soluble and water-insoluble drugs/NPs can be loaded into the core while maintaining high MRI contrast (Liu et al., 2017a). In magnetic liposomes synthesized by Yang et al. γ-Fe_2_O_3_ were encapsulated by liposomes doped with anethole ditholethione (Liu et al., 2017b). MR imaging was used to follow the accumulation of the magnetic liposomes in the tumor. Additionally, ultrasound was used to produce microbubbles (H_2_S) in order to ablate the tumor tissue via an increase in size (Liu et al., 2017b).

### Magnetic Microcapsules

Magnetic multilayer microcapsules composed of poly(allylamine hydrochloride) and poly(sodium 4-styrenesulfonate) and prepared by LbL deposition were utilized as magnetic delivery vehicles *in vitro* and *in vivo* (Voronin et al., 2017) *in vivo* experiments performed on mesenteric vessels of white mongrel rats reveal that capsules can be successfully trapped by the magnetic field and even stay there after the magnetic field is turned off. This work validates the effective control of microcapsules in a blood flow, making them promising drug delivery systems with remote navigation by the external magnetic field.

**Figure 2:**
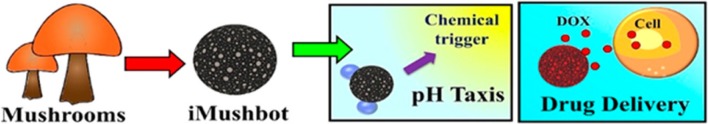
Schematic representation of the iMushbot action (Bhuyan et al., 2017). It is being reproduced with the permission of the copyright holder [American Chemical Society].

Microcapsules called iMushbots and prepared from mesoporous mushroom (*Agaricus bisporus*) fragments coated with magnetite NPs showed promising properties for targeted delivery (Bhuyan et al., 2017). The magnetite NPs played two roles (i) helping to pH-induced chemotaxis via the heterogeneous catalytic decomposition of the peroxide fuel in the presence of iron oxide and (ii) allowing a remote magnetic control of the iMushbot movement. The iMushbots also demonstrated higher drug retaining ability inside alkaline pH regions (human blood) and easy drug release in acidic medium (cancerous tissue) (Figure 2).

### Magnetic Fibers

Polyvinyl alcohol fibers filled with magnetite NPs were synthesized via infusion gyration and studied as biocompatible magnetically triggered drug delivery vehicles (Perera et al., 2018). The authors demonstrated that acetaminophen (model drug) uploaded via impregnation can be controllably released by magnetic actuation. Moreover, upon creating a magnetic field 90% cumulative release was observed in 15 min which was much higher than that without magnetic field. These results demonstrate a potential for remote delivery of drugs or other substances.

## Drug Uptake/Release

Uptake of drugs in magnetic drug delivery vehicles is carried out similar to non-magnetic carriers via conjugation (Chaudhary et al., 2015; Pourmanouchehri et al., 2018), hydrophobic interactions (Cho et al., 2018), absorption within porous structures (Kakar et al., 2013), etc. The drug release can be triggered by pH changes in the microenvironment (Wang et al., 2017; Wei et al., 2017; Wang, G. et al., 2018), by mechanical forces (Xu et al., 2018), NIR irradiation (Wang, Y. et al., 2018; Zheng et al., 2018), chemical reduction (Ao et al., 2018), HIFU (Moroz et al., 2001), and magnetic hyperthermia (Cho et al., 2018).

## Cytotoxicity

Cytotoxity of DOX bearing magnetic bioprobes has been discussed in a number of publications both for cancer cells and for healthy tissues (Lee et al., 2017; Ao et al., 2018; Sun, Q. et al., 2018). Cytotoxicity toward healthy cells is limited because most systems are localized and made biocompatible. It is demonstrated that the bioprobes without DOX do not kill cancer cells (Ao et al., 2018), while efficacy of magnetic bioprobes with DOX is comparable to that of free DOX (Ao et al., 2018). Cytotoxicity also increases with increasing amounts of DOX and/or upon NIR irradiation (Sun, Q. et al., 2018).

## Approaches to Magnetic Drug Targeting

### External Magnetic Field

In the majority of cases, the magnetic drug targeting/delivery is realized upon the application of an external magnetic field from electromagnetic coils (Hoshiar et al., 2017) or various types of permanent magnets (Carenza et al., 2014; Price et al., 2017; Shaw et al., 2017; Venugopal et al., 2017; Voronin et al., 2017; Shamsi et al., 2018). It was demonstrated that magnet geometry and tumor-magnet distance can be of crucial importance for the effective magnetic drug delivery (Shamsi et al., 2018; Wang, X. et al., 2018).

### Delivery Deep Inside the Body

Utilizing stationary external magnets to attract the magnetic drug carriers, it is difficult to target areas below 5 cm under the skin. A dynamic control of magnets to focus magnetic carriers to deep tissue targets has been proposed (Shapiro, 2009). Using first-principles magneto-statics and ferrofluid transport model, the author demonstrated that a sequence of actuations can push magnetic NPs through a center region, thus generating a focus at a deep target. In the other theoretical work, by rotating the magnet and setting a central axis to the target part, ferromagnetic drugs were accumulated in the target (Chuzawa et al., 2013). However, to the best of our knowledge no experimental studies have confirmed the conclusions of the theoretical work.

### Magnetic Implants

Instead of using an external magnetic field which could be problematic in the case of delivery to some organs, magnetic implants seem to be a viable alternative. Ge et al. reported targeted drug delivery to a biocompatible magnetic implant scaffold made of a magnetite/poly(lactic-co-glycolic acid) nanocomposite (Ge et al., 2017). In this case, a drug was attached to magnetic NPs to create a driving force for delivery. Such magnetic implants can be promising for a bone cancer, providing a precise cancer treatment. Magnetic implants can be imbedded in a fatty tissue to treat obesity (Saatchi et al., 2017) and in the inner ear to treat deafness (Le et al., 2017).

## Summary and Outlook

In summary, we can identify several essential rules which need to be followed for the development of successful magnetic drug delivery vehicles. The magnetic bioprobes need to be sufficiently large to possess high magnetization for efficient magnetic targeting. They have to allow for controlled drug uptake and release mechanisms to make them efficient delivery systems. Finally, they have to possess theranostic features (both diagnostic and therapeutic) to enhance the delivery and the drug action. The other key feature of promising magnetic drug delivery vehicles is long-circulating, stealthy systems which will not be cleared by a phagocyte system (Zhang et al., 2017, 2018). This can be realized by a combination of peptides and polymers in the particle outer shells. It is worth noting, however, that the degree of bioprobe sophistication is only warranted by the wealth of properties it delivers, as sometimes simpler systems can be quite satisfactory and less expensive.

It is worth noting that despite FDA approval and commercialization of iron oxide based bioprobes for MRI and hyperthermia, clinical trials of magnetic drug delivery received less attention. To the best of our knowledge, there were several small clinical trials (even in Phase III), but none resulted in FDA approval or commercialization (Wang et al., 2013; Min et al., 2015; Ulbrich et al., 2016).

We believe that the major unsolved problem in magnetic drug delivery is the absence of mechanisms for delivery into the depth of the body. The recent strategy of magnetic or magnetiziable implants seems to be promising but it requires a surgical intervention and in some cases cannot be implemented. Currently, efforts from physicists and engineers are required to move this field forward to real life applications.

## Author Contributions

PP carried out analysis of the literature and wrote a part on magnetic bioprobe synthesis. WM collected the literature and wrote the rest of the discussion on magnetic bioprobes. AA-G wrote part on approaches to magnetic targeting. LB wrote all other sections and oversaw the project.

### Conflict of Interest Statement

The authors declare that the research was conducted in the absence of any commercial or financial relationships that could be construed as a potential conflict of interest.
